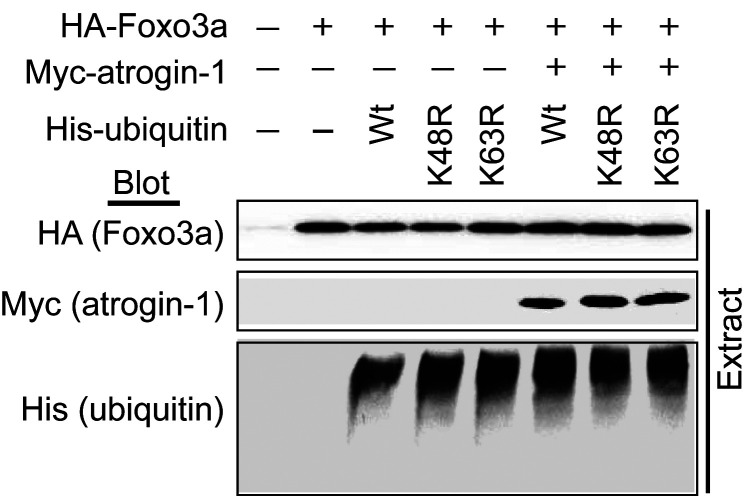# Atrogin-1 inhibits Akt-dependent cardiac hypertrophy in mice via ubiquitin-dependent coactivation of Forkhead proteins

**DOI:** 10.1172/JCI157373

**Published:** 2022-01-04

**Authors:** Hui-Hua Li, Monte S. Willis, Pamela Lockyer, Nathaniel Miller, Holly McDonough, David J. Glass, Cam Patterson

Original citation: *J Clin Invest*. 2007;117(11):3211–3223. https://doi.org/10.1172/JCI31757

Citation for this corrigendum: *J Clin Invest*. 2022;132(1):e157373. https://doi.org/10.1172/JCI157373

The authors recently became aware that the image for the HA (Foxo3a) blot in Figure 5C was a duplicate of the image presented as the anti-Myc (atrogin-1) blot in the right panel of Figure 4E. The corresponding authors have reviewed the original data and determined that the image in Figure 5C was incorrect. The corrected figure panel appears below. Additional lanes of the His (ubiquitin) blot, which were cropped from the original figure, are also displayed. The authors have stated that these errors were inadvertent and do not change the conclusions.

The authors regret the error.

## Figures and Tables

**Figure:**